# Novel risk model based on angiogenesis-related lncRNAs for prognosis prediction of hepatocellular carcinoma

**DOI:** 10.1186/s12935-023-02975-x

**Published:** 2023-08-07

**Authors:** Shicheng Xie, Jinwei Zhong, Zhongjing Zhang, Weiguo Huang, Xiaoben Lin, Yating Pan, Xiuyan Kong, Hongping Xia, Zhijie Yu, Haizhen Ni, Jinglin Xia

**Affiliations:** 1 Key Laboratory of Diagnosis and Treatment of Severe Hepato-Pancreatic Diseases of Zhejiang Province, The First Affiliated Hospital of Wenzhou Medical University, Wenzhou, 325000 China; 2 Department of Vascular Surgery, The First Affiliated Hospital of Wenzhou Medical University, Wenzhou, 325000 Zhejiang Province China; 3 Wenzhou Key Laboratory of Hematology, The First Affiliated Hospital of Wenzhou Medical University, Wenzhou, Zhejiang China; 4 Department of Interventional Radiology, The First Affiliated Hospital of Wenzhou Medical University, Wenzhou, 325000 China; 5 Liver Cancer Institute, Zhongshan Hospital, Fudan University, Shanghai, 200032 China; 6 School of Medicine & Advanced Institute for Life and Health, Zhongda Hospital, Southeast University, Nanjing, 210009 China

**Keywords:** Hepatocellular carcinoma, lncRNA, Machine learning, Angiogenesis, MIR210HG

## Abstract

Hepatocellular carcinoma (HCC) is a major cause of cancer-related death due to early metastasis or recurrence. Tumor angiogenesis plays an essential role in the tumorigenesis of HCC. Accumulated studies have validated the crucial role of lncRNAs in tumor angiogenesis. Here, we established an angiogenesis-related multi-lncRNAs risk model based on the machine learning for HCC prognosis prediction. Firstly, a total of 348 differential expression angiogenesis-related lncRNAs were identified by correlation analysis. Then, 20 of these lncRNAs were selected through univariate cox analysis and used for in-depth study of machine learning. After 1,000 random sampling cycles calculating by random forest algorithm, four lncRNAs were found to be highly associated with HCC prognosis, namely LUCAT1, AC010761.1, AC006504.7 and MIR210HG. Subsequently, the results from both the training and validation sets revealed that the four lncRNAs-based risk model was suitable for predicting HCC recurrence. Moreover, the infiltration of macrophages and CD8 T cells were shown to be closely associated with risk score and promotion of immune escape. The reliability of this model was validated by exploring the biological functions of lncRNA MIR210HG in HCC cells. The results showed that *MIR210HG* silence inhibited HCC growth and migration through upregulating PFKFB4 and SPAG4. Taken together, this angiogenesis-related risk model could serve as a reliable and promising tool to predict the prognosis of HCC.

**Supplementary Information:**

The online version contains supplementary material available at 10.1186/s12935-023-02975-x.

## Introduction

Hepatocellular carcinoma (HCC) ranks the 6th most common malignancies and the 3rd cancer-related death worldwide [1]. Early metastasis and high recurrence post-surgery contribute to the poor prognosis of HCC [2]. The rich vascular network within liver tissues is available for the angiogenesis of HCC, playing an essential role in promoting hepatic tumorigenesis and cancer progression [3, 4]. Although antiangiogenic drugs such as sorafenib and bevacizumab exhibit certain effect on partial HCC patients, they are still encumbered by the unsatisfied response rates and resistance [5, 6]. Hence, it is paramount to finding new angiogenic biomarkers for predicting prognosis of HCC patients.

Long non-coding RNAs (lncRNAs) are a family of RNAs with length exceeding 200 nucleotides and are capable of no or weak protein-coding capacity [7]. Multiple evidences have indicated that lncRNAs are critically involved in regulating diverse pathophysiological processes, including cell proliferation, apoptosis, drug resistance, and metastasis [8]. Particularly, recent studies reveal that lncRNAs are key modulators for angiogenesis. For example, lncRNA MYLK-AS1 promotes angiogenesis by targeting miR-424-5p/E2F7 axis and activating VEGFR-2 signaling pathway in HCC [9]. LncRNA RAB11B-AS1 induced by HIF-2 facilitates hypoxia-mediated angiogenesis and cancer metastasis [10]. Similarly, linc-ROR promotes the progression and angiogenesis of HCC via modulating DEPDC1 expression [11]. However, the explicit molecular mechanisms of angiogenesis lncRNAs in HCC remain poorly understood.

Herein, the integrated bioinformatics analysis was performed to verify four angiogenesis-related lncRNAs. Then, an angiogenesis-related lncRNAs risk model was developed for accurately prognostic prediction in HCC patients. In addition, the biological functions underlying the lncRNA signature were explored via *in-vitro* experiments. Hence, lncRNA MIR210HG was chosen to verify the accuracy of the model. The results demonstrated that MIR210HG could facilitate the tumorigenesis and angiogenesis of HCC via upregulating the expression of mRNA PFKFB4 and SPAG4. Therefore, the present angiogenesis-related multi-lncRNAs risk model is reliable for liver cancer survival prediction.

## Materials and methods

### Dataset acquisition and preprocessing

A total of 374 HCC patients’ transcriptome profiles, somatic mutation data (type: VarScan2 Variant Aggregation and Masking) CNV files and clinicopathological information were collected from the TCGA database [12]. Normalized and log2 transformation were conducted on gene expression data for subsequent analysis. Samples without essential clinicopathological information were excluded. The study was implemented on published TCGA guidelines.

### Angiogenesis associated lncRNAs

Genes involved in angiogenesis-related signaling pathways were obtained from HALLMARK and the GO database of GSEA (http://gsea-msigdb.org/gsea/index.jsp). The angiogenesis genes expression matrix was obtained from TCGA-LIHC database, including 418 angiogenesis- related mRNAs. A correlation test was conducted on the available angiogenesis-related genes and lncRNAs. The angiogenesis-related lncRNAs were defined as the lncRNAs with absolute value of correlation ≥ 0.4 and *p* value < 0.001. A network containing the lncRNAs and mRNAs was plotted and visualized by Cytoscape.

### Differential gene

The “limma” package was used in R to identify differentially angiogenesis-related lncRNAs. The lncRNA with Log_2_|FC| > 1, FDR < 0.05 were considered as significant difference. The volcano plot and heat map were conducted separately using “pheatmap” and “vioplot” packages in R.

### Clustering analysis and survival and recurrence analysis

The differentially expressed lncRNAs were analyzed by using “factoextra” package for unsupervised clustering. The samples were divided into three categories (vascular H, vascular M and vascular L) by the K-MEANS method. Patients’ OS and RS were analyzed using KM methods and plotted using the “survminer” package [13]. The log-rank test was employed to compare survival differences with *p* value < 0.05.

### Machine learning

LncRNAs were selected by univariate cox regression with *p* value < 0.001 for subsequent mechanical learning. Td-ROC analysis was used for evaluating clinical variables and the performance of classifier [14]. The AUC of td-ROC exhibited the predictive accuracy, and differences with *p* < 0.05 was considered statistically significant [15].

### Evaluation of immune cell infiltration

CIBERSORT algorithm, following as previously described [16], was utilized to visualize the proportion of infiltrating immune cells in RNA sequencing data. The proportion of immune cell subsets in HCC was compared by Wilcox test. Additionally, “immunedeconv” package was used to access immune-infiltration data from CIBERSORT, EPIC, quanTIseq, TIMER, xCell, and MCPCOUNTER databases. The correlation analysis was performed with risk scores [17].

### Gene set enrichment analysis

LncRNAs were divided into two groups by median: high-risk and low-risk groups. To clarify the specific molecular mechanism of these lncRNAs in HCC, GO and KEGG analyses were performed to compare the difference between high-risk and low-risk groups [13]. It was considered as significant differences in gene enrichment when | NES |≥ 1, FDR *q*-value < 0.25 and NOM *p* value < 0.01.

### Effective drug experiment

To identify efficient antivascular drugs, “pRRophetic” package was used. The efficient drugs were selected and reserved as candidate drugs for further screening. Meanwhile, NCI60 database was used to predict the efficiency of lncRNA targets.

### Cell culture

Human HCC cell lines (Huh7 and PLC/PRF/5) were purchased from the Cell Bank of the Chinese Academy of Science (Shanghai, China). All cell lines were cultivated in high glucose-DMEM containing 10% FBS (Gibco, USA) and 1% Pen-strep solution (Invitrogen, UK). Human normal liver cell lines (LO-2) were purchased from Cell Bank of the Chinese Academy of Science (Shanghai, China) and cultured in high glucose-DMEM containing 10% FBS (Gibco, USA) and 1% Pen-strep solution (Invitrogen, UK). HUVEC cells were purchased from Shanghai Institute of Cell Biology (Shanghai, China) and cultured in endothelial cell medium. All cells were cultured in a humidified 37℃ incubator filled with 5% CO_2_ and then passaged when the confluence reached 90%.

### Cell transfection

The sequences of siRNA targeting MIR210HG (si-MIR210HG) and matched control were provided by Sangon Biotech (China). Lipofectamine RNAiMAX (Invitrogen, USA) was used to transfect siRNAs into the HCC cells according to the manufacturer’s instructions. The si-MIR210HG sequence was listed below: 5’-GGGAUUUGGUUCACCUGAATT-3’.

Short hairpin RNA (shRNA) lentivirus for MIR210HG knockdown (sh-MIR210HG) and sh-NC lentivirus for Negative Control knockdown were purchased from GenePharma (Shanghai, China). The cells were transfected at an MOI of 20 and puromycin was used to select the stably transfected cells. The sh-MIR210HG sequence was listed below: 5’-CCCACUUGGCCUAUGCAUUTT-3’.

### CCK-8 assay

Cells were plated into 96-well plates at a density of 5000 cells/well. After cultured for a set of predefined time (24, 48, 72 h), 10 µl CCK-8 reagent was added into each well and incubated for further 2 h. The OD value at 450 nm was measured by a microplate reader (Thermo Fisher Scientific, USA).

### Colony formation assay

Cells were seeded at the density of 3000 cells/well into 6-well plates and cultured for 2 weeks. After washing with PBS, cells were fixed by 4% paraformaldehyde and stained with crystal violet. The colonies were captured and counted under a microscope (Leica, Japan).

### 5-Ethynyl-2′-deoxyuridine (EdU) staining

The EdU Kit (RiboBio, China) was used to evaluate the proliferation of cells according to the instructions from the manufacturer. In brief, cells were plated into 24-well plates with 1 × 10^5^ cells/well and maintained for 24 h. After the incubation with EdU solution for 2 h, cells were fixed with 4% paraformaldehyde and stained with Apollo solution. The proliferating cells were recorded under an inverted fluorescence microscope (Nikon, Japan). ImageJ software was applied to count the proliferated cells.

### Wounding healing assay

First, the Ibidi® Culture Insert Chamber (Ibidi®, German) was placed into the 6-well plates. Then, a total of 5 × 10^5^ cells were plated into the chamber of each well. After 24 h cultivation, the chambers were removed and approximately 500-µm wide wound was left on plates. Images were recorded by microscope at 0, 24 and 48 h. Then, ImageJ software was used to calculate the migrated distance of cells.

### Transwell assay

Totally, 3 × 10^4^ HCC cells in 200 µl medium (2% FBS) were added into the upper chamber (Corning, USA), while 600 µl medium containing 20% FBS was added in the lower chamber. After 24 h, the chambers were fixed by 4% paraformaldehyde. An optical microscope was used to photograph the migrated cells stained with 0.1% crystal violet.

### Tubule formation assay

Matrigel with reduced growth factors (Corning, USA) was slowly thawed at 4 °C. Then, 250 µl of Matrigel was added into 24-well plates and polymerized at 37 °C for 30 min. Then, HUVECs were resuspended in the CM of HCC cells and seeded at a density of 1 × 10^6^ cells/well onto the Matrigel. After incubating for 6 h at 37 °C, the formed tubules were observed under an optical microscope and counted using ImageJ software.

### RT-qPCR

The RNA Simple Total RNA Kit (TIANGEN, DP419) was used to extract total RNA from the HCC cells. The extracted RNAs were transcribed into cDNA using ReverTra Ace qPCR RT Kit (TOYOBO, China). RT-qPCR was performed on LightCycle96 (Roche, Switzerland) using SYBR Green. The relative RNA level was quantified by using 2 − ΔΔCt method and standardized to GAPDH. The primer sequences were listed in **Table **1. This study related to human specimens was approved by the ethical committee of the First Affiliated Hospital of Wenzhou Medical University (KY2023-R085). Informed consent was obtained from each subject for the sample collection and analysis.

**Table 1:** RT-qPCR primer sequences

**Primer**	**Sequence (5’-3’)**
MIR210HG	F: GCTTGGTAGAGTGTCACGCC R: CATCTGACCGAGCCAGTTTG
GAPDH	F: CTCTGCTCCTCCTGTTCGAC R: ACCAAATCCGTTGACTCCGA

### Western blotting

Appropriate amount of RIPA (contained PMSF) was used to extracted total protein. After separated on a 10% SDS-PAGE gel, proteins were transferred onto a PVDF membrane (Millipore, USA). After blocked by 5% BSA (Beyotime, China), the membrane was incubated with the primary antibody overnight at 4 °C. Then, the membrane was washed and incubated with the secondary antibody for 1 h at room temperature. After washing, the bands were visualized using VisionWorks (Analytik Jena, Germany). ImageJ software was used to quantify the intensity of the protein bands. Antibodies used in this study were listed as following: rabbit anti-human PFKFB4 (1:1000, HuaBio, China), rabbit anti-human SPAG4 (1:1000, Proteintech, China), rabbit anti-human β-Catenin (1:1000, CST, USA), rabbit anti-human SNAIL (1:1000, CST, USA), rabbit anti-human Claudin-1 (1:1000, CST, USA), mouse anti-human β-Actin (1:1000, Abcam, USA), horse anti-mouse IgG, HRP-linked antibody (1:5000, CST, USA), and goat anti-rabbit IgG, HRP-linked antibody (1:5000, CST, USA).

#### Tumor xenografts in nude mice

All male BALB/c nude mice (4–6 weeks) used in this study were purchased from Charles River (Beijing, China). All animal experiments were conducted in accordance with Laboratory Animal Ethic Committee of the First Affiliated Hospital of Wenzhou Medical University (WYYY-AEC-2023-025). A total of 5 × 10^6^ cells mixed with Matrigel (1:1) were subcutaneously injected into left flank of each mouse. After 5 days following the injection, the tumor sizes and body weight of mice were monitored with a caliper every 3 days. The tumor volume was calculated (in mm^3^) as length × width × width/2.

#### Immunohistochemistry

For IHC, xenograft tissues were fixed in 4% paraformaldehyde, dehydrated, paraffin-embedded, and cut into sections. Then, the sections were incubated with a Ki-67 antibody (1:300, Servicebio, China) and then incubated with horseradish peroxidase (HRP)-conjugated goat anti-rabbit IgG (1:200, Servicebio, China). Images were obtained by a light microscope (Olympus, Tokyo, Japan).

#### Statistical analyses

All statistical analysis was performed using GraphPad Prism 8 (GraphPad). The variations of data were evaluated as the means ± SD. Two-tailed Student t test was applied for comparison of continuous variables. When group numbers were more than two, One-way ANOVA analysis was performed to determine the difference. P < 0.05 was defined as statistical significance.

## Results

### Identification and hierarchical clustering analysis of angiogenesis-related lncRNA

**Fig. 1:**
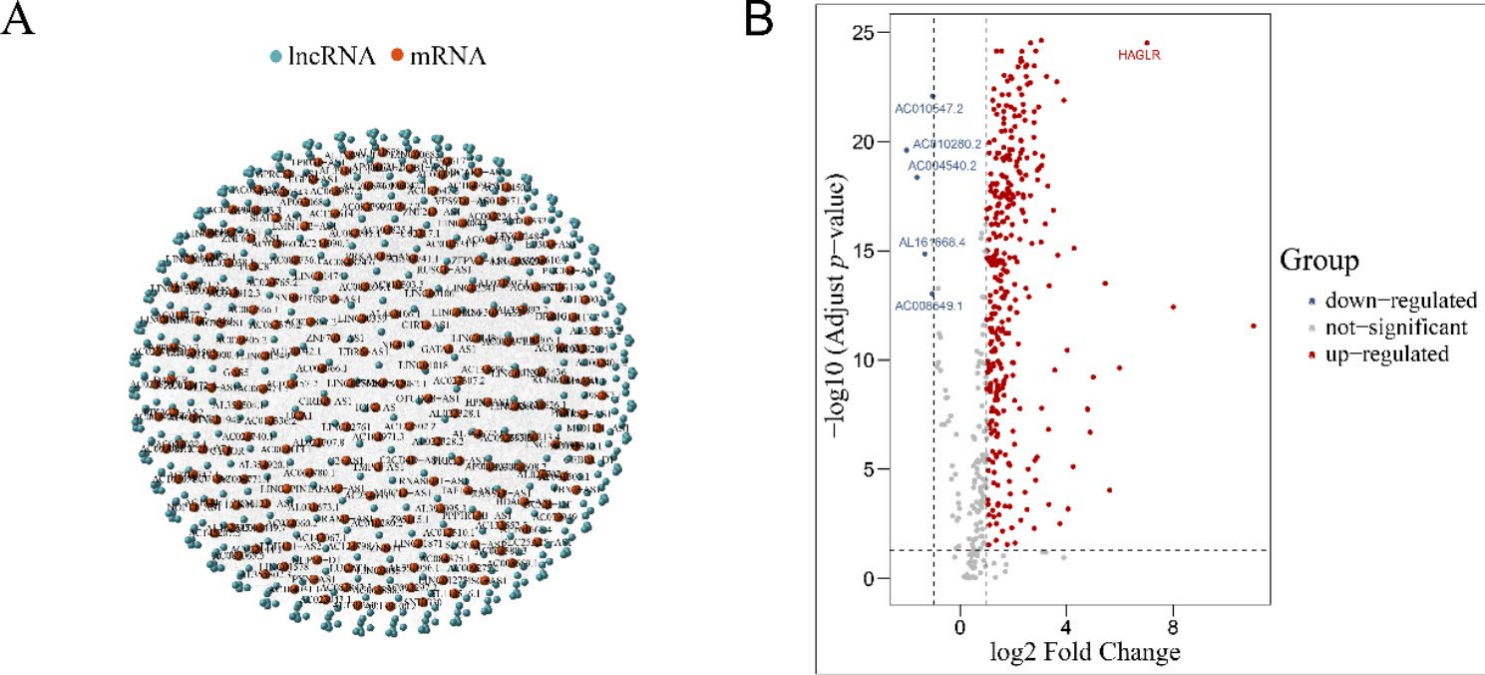
Identification of angiogenesis-related lncRNA. (**A**) The interaction diagram of angiogenesis-related mRNAs and lncRNAs; (**B**) The differential expression of vascular-related lncRNAs in tumor and adjacent tissues. |log2FC|>=1, FDR < 0.05 means significant difference

**Fig. 2:**
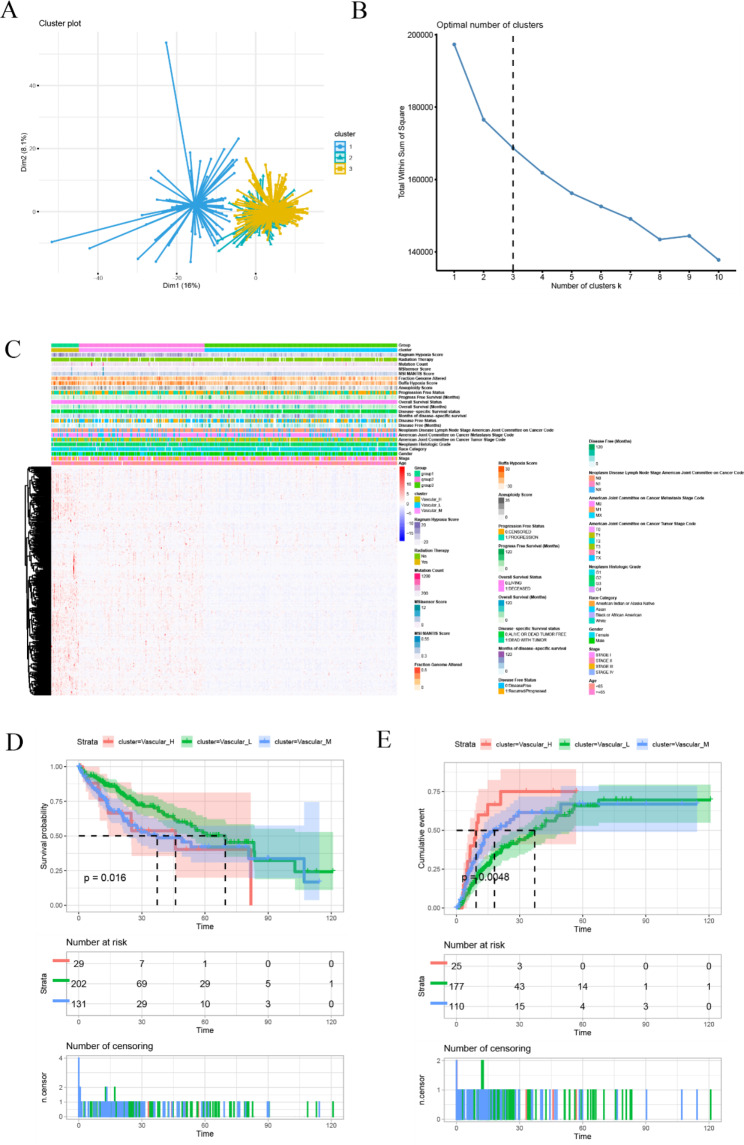
Angiogenesis-related lncRNAs identify distinct HCC subgroups with different recurrence status. (**A-C**) Three distinct groupings, vascular_H, vascular_M, and vascular_L, were generated by hierarchical clustering analysis based on the expression levels of angiogenesis-related lncRNAs in TCGA; (**D**, **E**) KM survival curves of different angiogenic lncRNA isoforms in TCGA, overall survival and time to recurrence, respectively. The vascular_H subgroup had a worse prognosis than the vascular_L subgroup (OS: log-rank *p* = 0.016; Recurrence: log-rank *p* = 0.0048)

To identify prognosis-related lncRNAs in HCC, we first obtained the LIHC project from the TCGA dataset with a total of 360 HCC patients. Then, we performed 418 angiogenesis-related mRNAs on the hallmark and GO datasets in which 379 mRNAs have related genes expressed in the TCGA-LIHC dataset. Finally, through correlation analysis of mRNAs and lncRNAs, a total of 529 angiogenesis-related lncRNAs were obtained (Fig. 1A). To explore the differentially expressed lncRNAs in tumor and adjacent tissues, the limma package was used. Totally, 343 up-regulated lncRNAs and 5 down-regulated lncRNAs were obtained (Fig. 1B). Hierarchical clustering analysis showed that the expression patterns of 348 lncRNAs were able to distinguish the prognosis of HCC patients. According to the unsupervised clustering of 348 lncRNAs expression matrices, 360 HCC patients were clustered into Vascular_H, Vascular_M and Vascular_L clusters (Fig. 2A-C). Subsequently, KM survival analysis showed that OS and recurrence rates were different between the three groups. As shown in Fig. 2D, although there was no significant difference between Vascular_H and Vascular_M groups due to inadequate quantity of Vascular_H group, Vascular_L group exhibited a better OS rate than Vascular_H and Vascular_M groups. Meanwhile, for the analysis of recurrence rate, Vascula_H and Vascular_M groups had higher recurrence rates than Vascular_L group, respectively (Fig. 2E). These results indicated that these grouping based on angiogenesis-related lncRNAs could effectively predict the prognosis of HCC patients.

### Establish classification model based on angiogenesis-related lncRNA

**Fig. 3:**
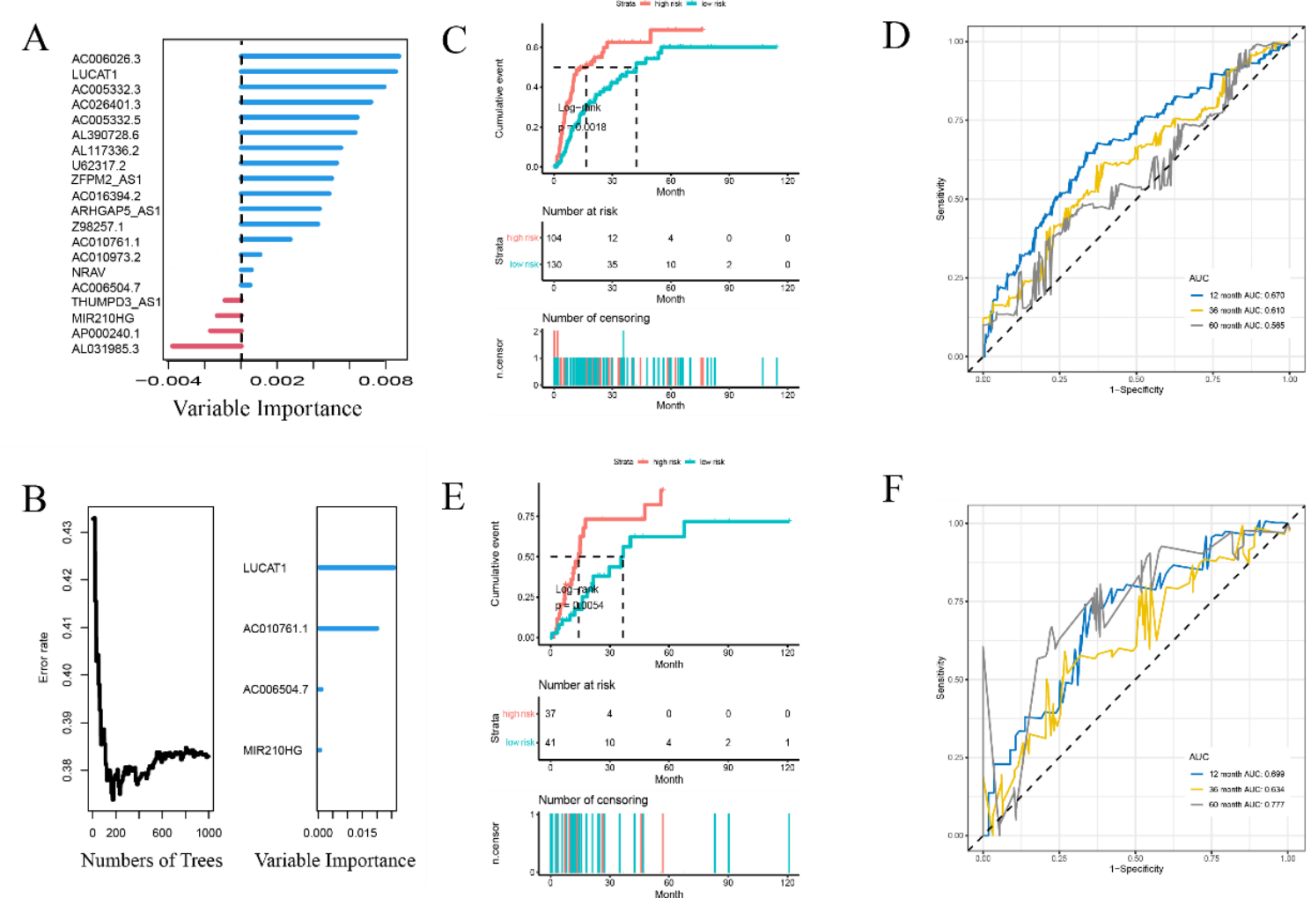
Machine learning builds a recurrence prediction model based on angiogenesis-related lncRNA. (**A**) Twenty vascular-related lncRNAs were significantly associated with recurrence by univariate cox analysis, with a *p* value of less than 0.001 as the inclusion criterion; (**B**) Random survival forest analysis identified 4 key lncRNAs in TCGA, namely LUCAT1, AC010761.1, AC006504.7, and MIR210HG; (**C, E**) KM survival curve validation of predictive effect in training cohort; (**C**) and validation cohort (**E**); (**D, F**) Time-dependent ROC curves of 4-lncRNAs-based classifiers; time-dependent ROC curves of multiple lncRNA-based risk models for recurrence

To explore the angiogenesis-related lncRNAs-based classifier for HCC prognostic prediction, the univariate cox analysis was performed on 348 differentially expressed lncRNAs and the lncRNAs with *p* value < 0.001 were selected for further study. As indicated in Fig. 3A, a total of 20 lncRNAs were obtained and used for in-depth study of machine learning. Next, the random forest algorithm was performed to further screen the 20 lncRNAs. The seed was set to 123, and patients were randomly divided into the training and validation sets at a 3:1 ratio. The random Forest SRC package was used to construct random forest survival model, which is the R/Bioconductor package performed using R (version 3.6.1). After 1,000 random sampling cycles calculated by the random forest algorithm, four lncRNAs were highly associated with HCC prognosis, namely LUCAT1, AC010761.1, AC006504.7 and MIR210HG (Fig. 3B). Then, the multi-lncRNAs classifier was constructed based on the weight of these four lncRNAs to score each HCC patient. Moreover, the patients were separated into high- and low-risk groups by the classifier with the median risk score of 33.578 as the optimal cutoff threshold.

### Evaluation and validation of the multi-lncRNAs classifier

**Fig. 4:**
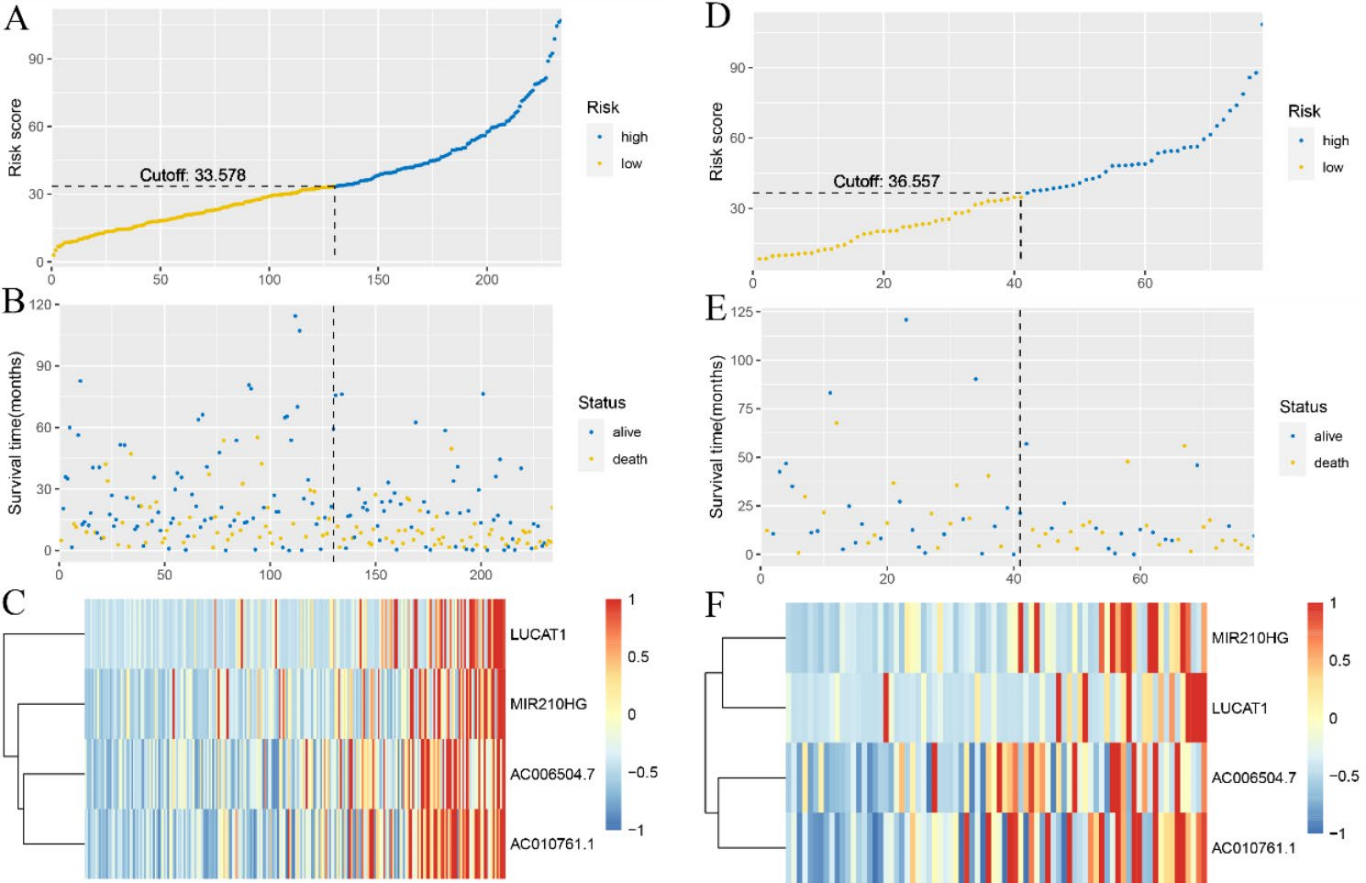
Assessment of multiple lncRNA-based risk models. (**A-F**) Distribution of survival status and risk score in training set (**A, B**) and test set (**D, E**), respectively. Heatmap of expression differences between high- and low-risk groups in the training set (**C**) and test set (**F**)

To evaluate the predictive accuracy of the multi-lncRNAs classifier on recurrence rate of HCC patients, KM and td-ROC curve analyses were applied. As shown in Fig. 3C and E, there was significant difference between high- and low-risk groups on HCC recurrence in both the training and validation sets by KM analysis. Rd-ROC curve analysis also indicated substantially prognosis predictive accuracy of the classifier that the AUC scores was 0.670, 0.610, 0.565 in the training set and 0.699, 0.634, 0.777 in the validation set at 12, 36 and 60 months, respectively (Fig. 3D F). As shown in Fig. 4, the distribution of survival status, OS time, and lncRNA expression were separately displayed due to the median risk score as the cutoff threshold in the training and validation sets. This indicated that HCC patients with higher scores had more relapse tendencies. Overall, this classifier based on angiogenesis-related lncRNAs was reliable and suitable for predicting HCC recurrence.

### Immune cell infiltration associated with the risk score

**Fig. 5:**
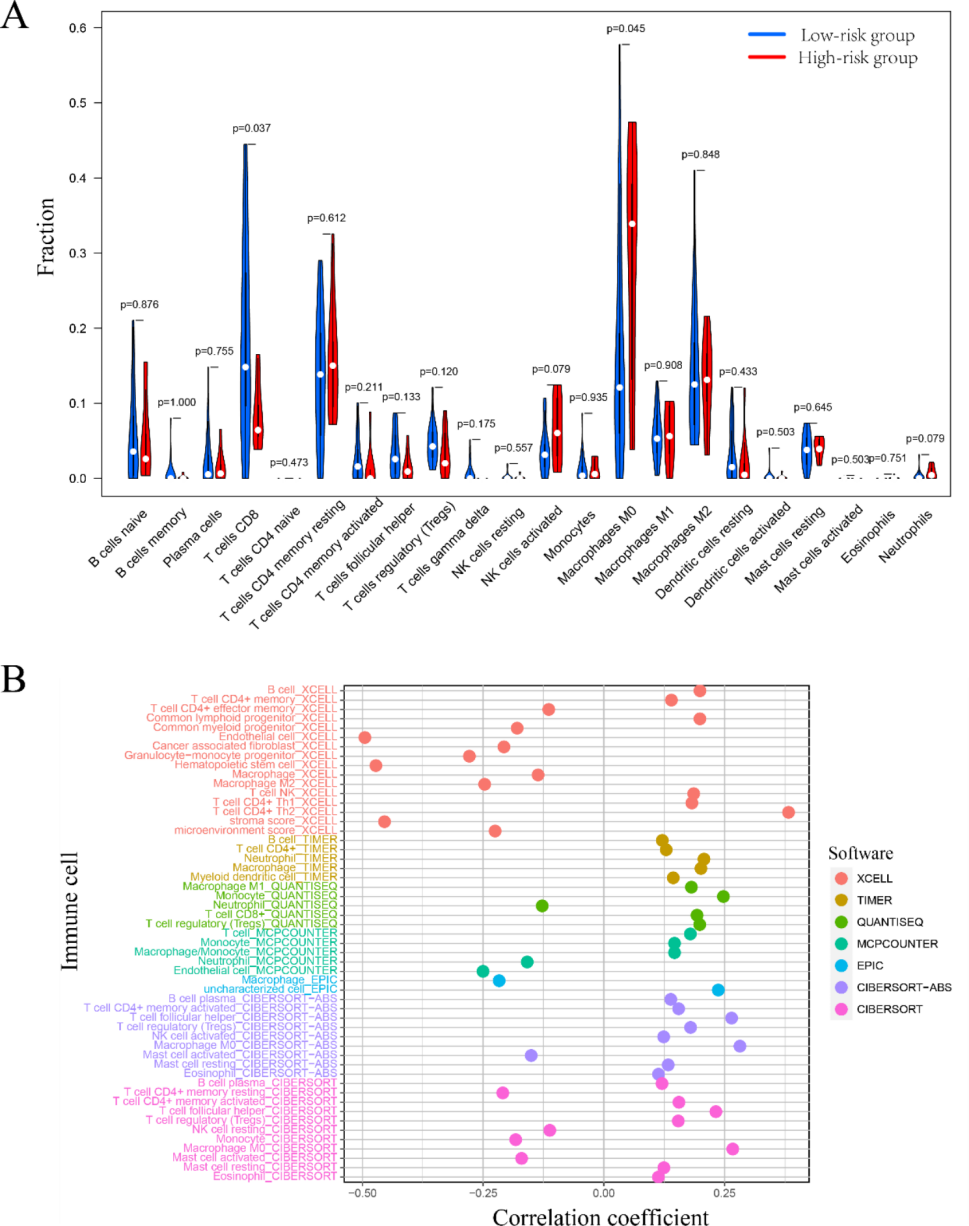
Immunological assessment of high- and low-risk groups. (**A**) The composition of 22 immune cells for the two clusters calculated by the CIBERSORT method; (**B**) Risk model correlates with multiple immune cells. *p* < 0.05

It is generally believed that tumors have a large number of tumor angiogenesis, which could secrete various chemokines to recruit immune cells. Particularly, changes in tumor microenvironment and different immune cell infiltration would significantly influence the prognosis of cancer patients. Therefore, the immune infiltration between high- and low-risk groups was investigated by the CIBERSORT algorithm. As indicated in Fig. 5A, the high-risk group had more M0 macrophages infiltration (P = 0.045, Wilcox test), while the low-risk group had more CD8 T cells infiltration (P = 0.037, Wilcox test). Additionally, we summarized the status of HCC immune infiltration by different databases, including XCELL, TIMER, QUANTISEQ, MCPCOUNTER, EPIC, and CIBERSOR (Fig. 5B). The results revealed that CD4 + effector memory T cells, common myeloid progenitor cells, endothelial cells, cancer associated fibroblasts, granulocyte-monocyte progenitor cells, hematopoietic stem cell, macrophages, macrophages M2, stroma score and microenvironment score exhibited negative correlations to the risk score. Conversely, B cells, T cell CD4 + memory cells, common lymphoid progenitor cells, NKT cells, T cell CD4 + Th1 cells and T cell CD4 + Th2 cells were positively related to the risk score. Subsequently, the results also revealed the positive correlation between M1 macrophage infiltration and risk score in each database separately, which showed negative correlation in M2 macrophage, as shown in **Figure** S1.

### GSEA

The biological functions of the lncRNAs were related to the predictive performance of risk models based on four angiogenesis-related lncRNAs in HCC. To explore the potential mechanism, we performed GSEA based on GO database and KEGG pathways in the high- and low-risk groups. The top 10 of significantly enriched pathways were recorded, as showed in **Figure** S2. The results of GO analysis revealed that these lncRNAs were mainly involved in regulating cellular amino acid metabolism, nuclear RNA metabolism, nuclear RNA processing, and protein activation cascade. Additionally, KEGG pathway functional enrichment analysis showed the consistent enrichment of base excision-repair, cell cycle, complement and coagulation cascade, fatty acid metabolism, primary bile acid biosynthesis, glycine serine and threonine metabolism, pyrimidine metabolism, RNA degradation, splicing body, valine leucine and isoleucine degradation.

### Target prediction and drug screening

**Fig. 6:**
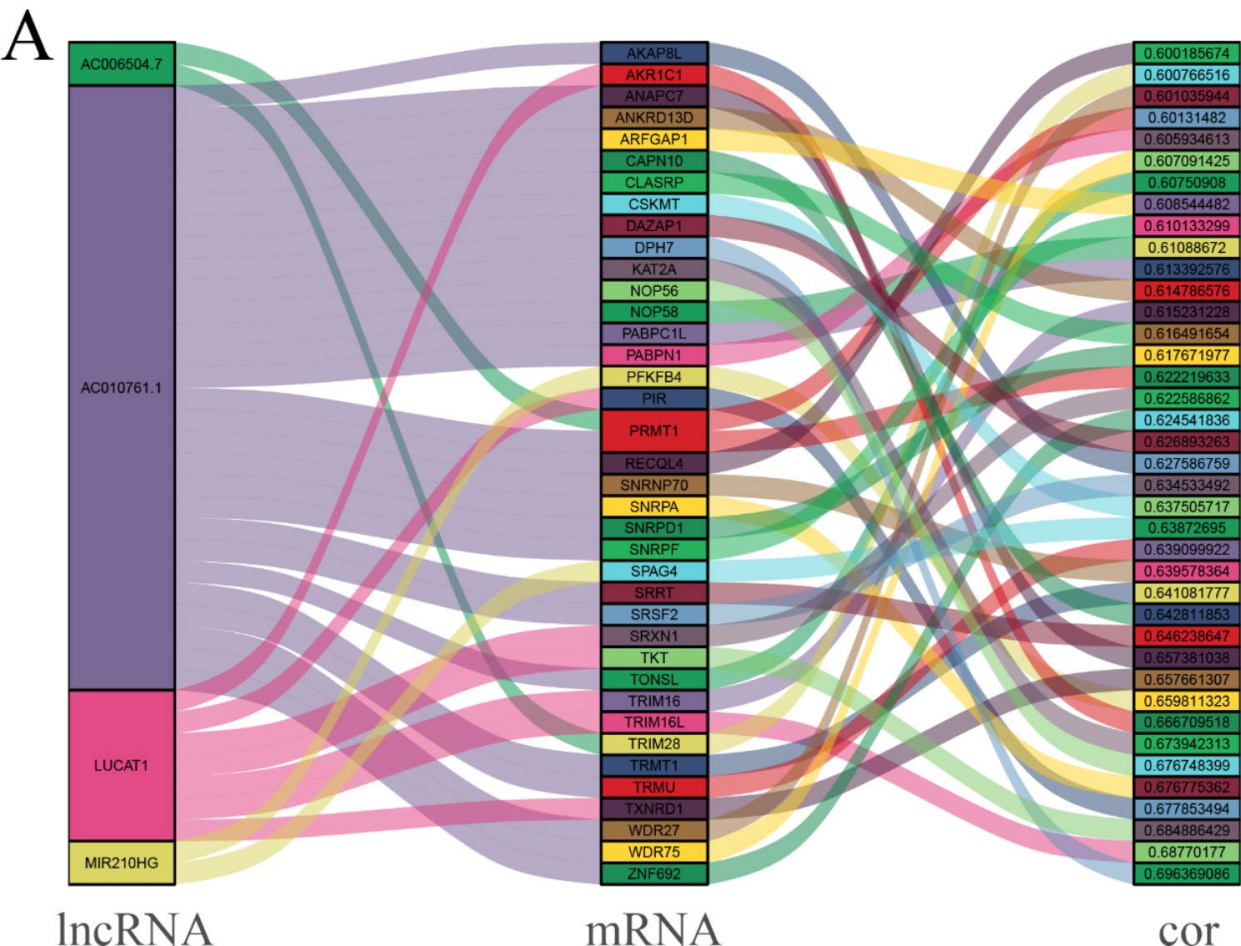
Potential pathways of angiogenesis-related lncRNAs. (**A**) Correlation analysis and co-expression network of vascular-related lncRNAs and mRNAs.

To further explore the potential molecular mechanism of HCC occurrence, these four lncRNAs for mechanical learning were used as potential prediction targets. The mRNAs with possible regulatory relationships were identified through correlation analysis. The results revealed that these four lncRNAs targeted a total of 40 mRNAs (Fig. 6), which may be involved in the underlying mechanisms of HCC tumor angiogenesis and metastasis. In addition, we conducted drug sensitivity analysis of HCC patients to screen suitable antivascular drugs using IC_50_ database. As shown in **Figure** S3, HCC patients in high-risk group were more sensitive to antivascular drugs such as A.770,041, AICAR, AKT.inhibitor.VIII, while patients in low-risk group were more sensitive to ABT.888, AG.014699, ATRA. Subsequently, we predicted the therapying target to lncRNAs of LUCAT1, AC010761.1, AC006504.7 and MIR210HG using CellMiner database through sequencing data of NCI60. The sequencing data of multiple drug trials of LUCAT1 and MIR210HG was shown in **Figure** S4.

### MIR210HG facilitates proliferation and metastasis of HCC cells in vitro and vivo

**Fig. 7:**
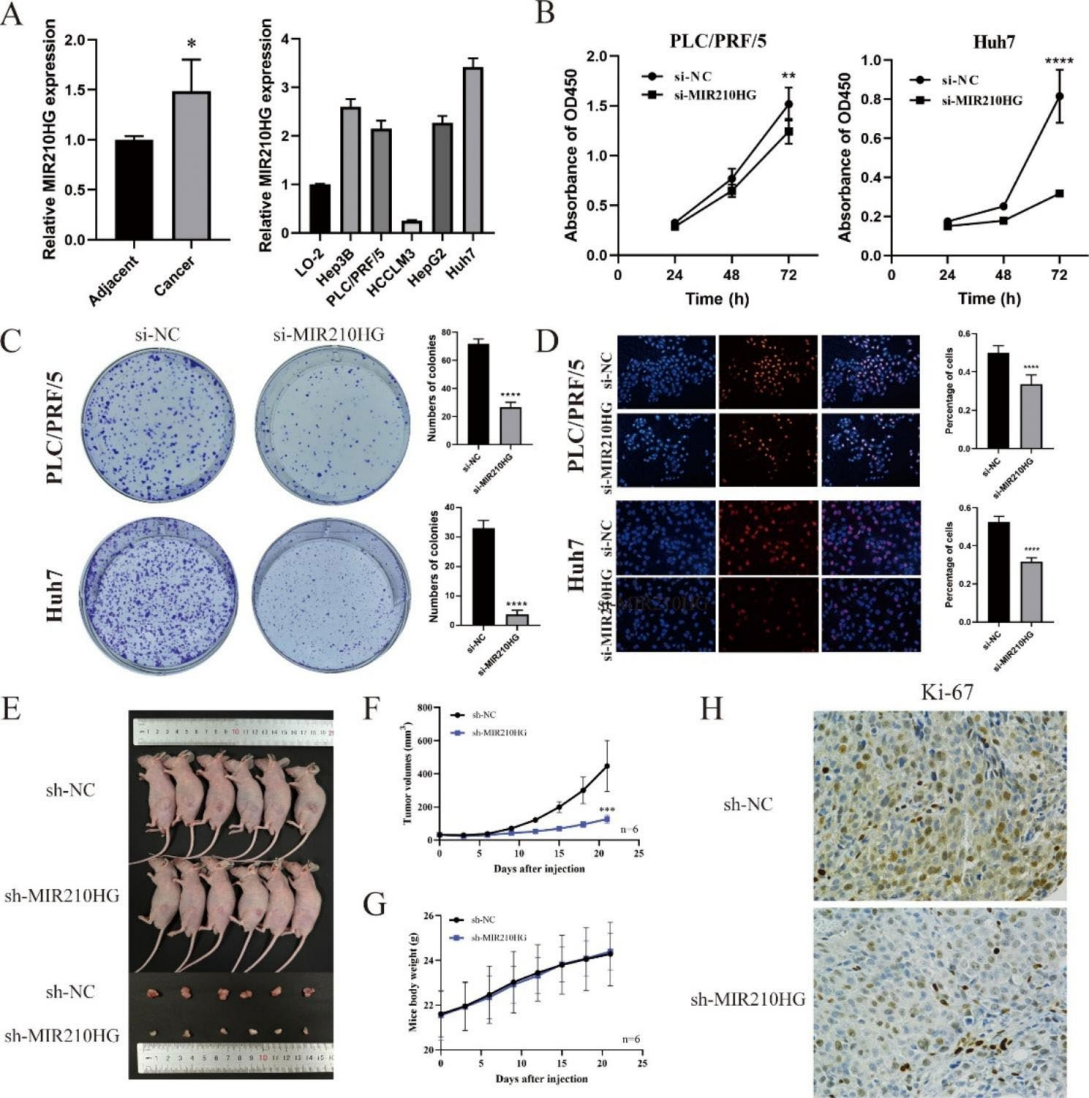
MIR210HG promoted HCC cells proliferation. (**A**) RT-PCR analysis of the MIR210HG expression of HCC tissue and cell lines; (**B**) The CCK8 assay confirmed the function of MIR210HG on HCC cell proliferation; (**C, D**) The clone assay and EdU immunofluorescence staining were used to assess the effect of silencing *MIR210HG* on PLC/PRF/5 and Huh7 cells; Magnification: 100×. (**E**) Knockdown of MIR210HG inhibited Huh7 xenografts growth; (**F-G**) Tumor volumes and mice body weights were recorded every 3 days; (**H**) Immunohistochemistry analysis of Ki-67 protein levels in xenograft tumor tissues; the knockdown of MIR 210HG decreased the expression of Ki-67(×400)

To validate the function of lncRNA MIR210HG in HCC development, *MIR210HG* was successfully silenced in the PLC/PRF/5 and Huh7 cells by siRNAs (Figure S5A). PCR results found that the level of MIR210HG was significantly higher in HCC tissues and cell lines, compared with normal adjacent tissues and LO-2 cells (Fig. 7A). Preliminarily, CCK-8, EdU and colony formation assays were conducted to explore the effect of MIR210HG on HCC cells proliferation. The CCK-8 results revealed that the proliferation was dramatically inhibited in si-MIR210HG-PLC/PRF/5 and si-MIR210HG‐Huh7 cells as compared to the control groups (Fig. 7B). Consistently, the proliferation of HCC cells was significantly decreased si-MIR210HG-PLC/PRF/5 and si‐MIR210HG‐Huh7 cells, as evidenced by reduced colony formation (Fig. 7C) and EdU staining (Fig. 7D). To determine the growth-promotive role of MIR210HG in vivo, the MIR210HG-knocked Huh7 cells by shRNA were constructed and a tumor xenograft model was established using the cells. A set of shRNA sequences were employed to knock down MIR210HG in Huh7 cells. Results from PCR verified the success of MIR210HG silence in Huh7 cell line (Figure S5B). Therefore, a total of 5 × 106 sh-MIR210HG Huh7 cells (sh-MIR210HG) and control cells (sh-NC) were injected subcutaneously into the BALB/c nude mice (n = 6 mice per group). As is shown in Fig. 7E-F, the tumors developing from sh-MIR210HG Huh7 cells grew slower than those from sh-NC Huh7 cells. The body weights of mice in each group were not significantly different (Fig. 7G). Further immunohistology analysis also revealed lower Ki-67 protein levels in tumor tissues from mice with sh-MIR210HG Huh7 cells (Fig. 7H). These results indicated that MIR210HG could inhibit the viability and tumor growth of HCC cells both in vitro and in vivo.

**Fig. 8:**
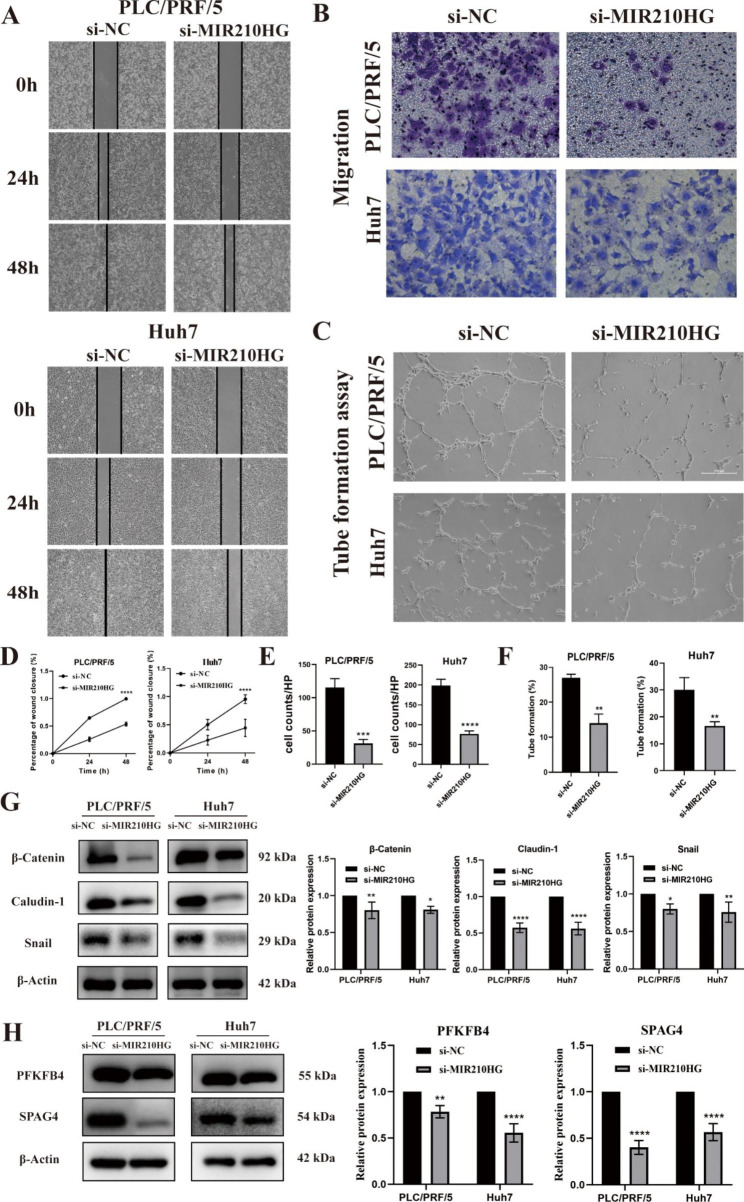
(**A, B**) Wound healing assay and transwell assay for analysis of migration after silencing *MIR210HG* on PLC/PRF/5 and Huh7 cells; (**C**) Analysis of vasculogenesis ability of HUVECs treating with si-MIR210HG-PLC/PRF/5 and si-MIR210HG-Huh7 conditional medium; Magnification: 100×; (**D-F**) Quantitative analysis of the wound closure (**D**), the migration rate (**E**), and tube formation (**F**); (**G**) Detection of epithelial mesenchymal transition-related protein levels of silencing *MIR210HG* cells by western blotting; (**H**) Western blotting analysis of PFGFB4 and SPAG4 protein expression after silencing *MIR210HG* on PLC/PRF/5 and Huh7 cells. The samples were derived from the same experiment and blots were processed in parallel

Importantly, the present bioinformatics analysis implied that lncRNA MIR210HG might be involved in promoting HCC metastasis. Therefore, the wound healing and transwell assays were performed to verify the migration-promoting ability of MIR210HG in HCC. Wound closure was remarkably suppressed in the si-MIR210HG-PLC/PRF/5 cells compared to the si-NC‐PLC/PRF/5 cells, with same performance in Huh7 cells (Fig. 8A and D). Consistently, the transwell assays revealed an obvious decrease in the migration capacity of si-MIR210HG-PLC/PRF/5 cells and si-MIR210HG-Huh7 cells, as compared to the control groups (Fig. 8B and E). In addition, the capillary network formation in HCC cells was detected after *MIR210HG* knockdown. Figure 8 C and 8 F showed that the tube formation of HUVEC cells was significantly inhibited when cultured in the CM of *MIR210HG*-silenced Huh7 and PLC/PRF/5 cells. Moreover, the EMT‐related markers expressions were detected by western blotting upon *MIR210HG* silencing (Fig. 8G**)**. Notably, our previous correlation analysis of lncRNAs and mRNAs showed that MIR210HG specifically targeted PFKFB4 and SPAG4. Strikingly, western blot revealed the expression of PFKFB4 and SPAG4 was remarkably decreased in si-MIR210HG-PLC/PRF/5 and si-MIR210HG-Huh7 cells compared to control groups (Fig. 8H).

## Discussion

HCC is a highly heterogeneous malignant tumor and often results in unstable therapeutic efficacy and inaccurate prognosis [18]. Generally, the progression of HCC is closely associated with genetic and epigenetic changes which may induce poor prognosis of HCC [19, 20]. Particularly, considering the importance of poor prognosis of HCC, identification of accurate and specific biomarkers for HCC is required. Tumor growth needs a complex system of blood vessels to provide oxygen and nutrients [21]. Angiogenesis is triggered by instability of the existing microvasculature, which results in the vascular permeable extracellular matrix remodeling and endothelial cells activation [22–24]. Then, the activated endothelial cells further grow and migrate to generate new blood vessels. Subsequently, pericyte is activated and recruited to stabilize new blood vessels [25]. During the process of angiogenesis, the amount of proangiogenic factor expression is balanced by the release of antiangiogenic factors [26]. As the cancer progression, tumor cells, vascular endothelial cells, immune cells and pericytes produce excessive proangiogenic factors, such as VEGF [27], bFGF [28], and HGF [29, 30], to break this balance and lead to excessive activation and recruitment of endothelial cells and pericytes. As reported, the level changing of these proangiogenic factors in HCC patients could also significantly regulate the process of HCC. For example, increased level of VEGF after surgical, RFA or TACE treatment predicts poor prognosis in HCC patients [31]. Similarly, rapid recurrence of HCC is associated with higher level of PlGF, PDGF and ANG-2 [32, 33]. However, previous studies are limited in exploring angiogenic factors as potential prognostic biomarkers. Commonly, the mainstream biomarkers are based on genes and mRNAs, which play an important role in protein translation and synthesis. Interestingly, lncRNAs have special biological functions in participating various processes by targeting RNA, DNA and proteins [34, 35]. Emerging evidences have indicated that lncRNAs play a vital role in the progress of HCC. However, the attempt that using genetic characteristics to predict the prognosis of HCC patients is still limited.

In the present study, we established an angiogenesis-related risk model to predict the survival and recurrence in HCC patients. Compared with previous studies, our model possessed a series of advantages. First, a total of 360 HCC patients were involved in our study, in which 312 patients provided the follow-up data for recurrence. In addition, 14,087 lncRNAs were isolated from the LICH dataset in this study. Thus, these adequate sample size of patients and lncRNAs could apparently reduce the offset. Secondly, an appropriate method was developed to identify remarkable lncRNA markers in the high-dimensional dataset. A total of 529 angiogenesis-related lncRNAs were obtained for further analysis. Upon univariate cox analysis on these angiogenesis-related lncRNAs of HCC prognosis, 20 lncRNAs were selected for random forest algorithm. Subsequently, as highest recurrence correlation of HCC identified by random forest algorithm, lncRNA LUCAT1, MIR210HG, AC010761.1and AC006504.7 were utilized to establish classifier. As previously reported, LUCAT1 could promote tumorigenesis in HCC by inhibiting ANXA2 phosphorylation [36]. Wang Y et al. [37] found that MIR210HG played an oncogenic role in HCC. However, the molecular mechanism of MIR210HG driving progression of HCC is still uncleared. In addition, little is known about the role of AC010761.1 and AC006504.7 in HCC development and progression, which needs further study in the future.

The effectiveness of the predictive model mainly depends on the function of candidate lncRNAs because the risk score was calculated based on the expression of these lncRNAs. Hence, we selected lncRNA MIR210HG to preliminarily explore biological functions. The results showed that silencing *MIR210HG* inhibited cell growth and migration through upregulating PFKFB4 and SPAG4. As previously reported, LUCAT1 could also promote tumorigenesis in HCC. These data collectively suggest that the angiogenesis-related lncRNAs are important for tumor proliferation and migration. Thus, the risk model established by these lncRNAs exerts significant prognostic prediction value in HCC.

To further validate the accuracy of the established risk model, the change of immune cell infiltration associated with the risk score was compared. The results indicated that macrophages were closely associated with risk score, which might induce the angiogenesis and promote the immune escape in HCC. Meanwhile, the higher infiltration of CD8 + T cells in the low-risk group suggested that immune response helped reduce the risk of tumor recurrence. Moreover, GSEA indicated that the pathways were enriched in cellular metabolism, which might associate with the metabolic reprogramming of HCC. For precise treatment, drug sensitivity screening was conducted. The results may provide a guidance for designing therapeutic strategies individually.

However, there are some limitations in our study that need to be interpreted. First, the TCGA database is deficient in information of immunotherapy, confining further use of the risk model in predicting the immunotherapy response. Second, although our experimental data can support our findings, there still need additional experiments to further explore the specific mechanisms of these lncRNAs on HCC metastasis.

In conclusion, a novel and effective risk model was established using four angiogenesis-related lncRNAs. The risk model served as a powerful and reliable tool in predicting prognosis of HCC. Moreover, the risk model presented here has the potential for predicting the prognosis in other cancer types with rich blood supply such as gastric cancer. Indeed, highly expressions of both LUCAT1 and MIR210HG were also found in gastric cancer cells. Previous study has proved that LUCAT1 promotes the proliferation and metastasis of gastric cancer by regulating miR-134-5p/YWHAZ axis [38, 39]. Thus, the risk model in our study is conceptually new and its results can help to guide individual therapeutic strategy design.

### Electronic supplementary material

Below is the link to the electronic supplementary material.

**Figure S1**. Macrophage infiltration in high and low risk groups. (A) Boxplots of differential distribution of macrophages in high- and low-risk groups across various databases. p<0.05. **Figure S2**. Gene set enrichment analysis (GSEA) of angiogenesis-related lncRNA classifiers. (A) Gene set enrichment analysis of high- and low-risk groups in the GO database. (B) Gene set enrichment analysis of high and low risk groups in the KEGG database GSEA, gene set enrichment analysis. When | NES | ≥ 1, FDR q-value <0.25 and NOM p-value < 0.01 were considered significant. **Figure S3**. (a) The association between voltage and the droplet diameter when the flow rate was 100 μl min-1; (b) The association between flow rate and the droplet diameter when the voltage was 6 kV; (c) The association between the inner flow rate (F1) and the droplet diameter when the outer flow rate F2 was 8 ml h-1; (d) The association between F2 and the droplet diameter when F1 was 0.4 ml h-1. **Figure S4**. Drug sensitivity screening for LUCAT1 and MIR210HG. **Figure S5**. Relative MIR210HG expression after using si-MIR210HG and sh-MIR210HG.

## Data Availability

The datasets used and/or analysed during the current study are available from the cancer genome database (TCGA: LIHC) https://portal.gdc.cancer.gov.

## Abbreviations

ANG-2: Angiopoietin-2
AUC: Area under the curve
bFGF: Basic fibroblast growth factor
BSA: Bovine serum albumin
CST: Cell signaling technology
FBS: Fetal bovine serum
GO: Gene ontology
GSEA: Gene set enrichment analysis
HCC: Hepatocellular carcinoma
HGF: Hepatocyte growth factor
KEGG: Kyoto encyclopedia of genes and genomes
KM: Kaplan-Meier
LncRNAs: Long non-coding RNAs
MAF: Mutation annotation format
OD: Optical density
OS: Overall survival
PDGF: Platelet-derived growth factors
PFKFB4: Phosphofructo-2-kinase/fructose-2,6-biphosphatase 4
PlGF: Placental growth factor
RFA: Radiofrequency ablation
RS: Recurrence survival
SPAG4: Sperm‑ associated antigen 4
TACE: Transarterial chemoembolization
TCGA-LIHC: The Cancer Genome Atlas Liver Hepatocellular Carcinoma
td-ROC: Time-dependent recipient operation characteristics
VEGF: Vascular endothelial growth factor

## References

[1] Sung H, Ferlay J, Siegel RL, Laversanne M, Soerjomataram I, Jemal A, Bray F (2021). Global Cancer Statistics 2020: GLOBOCAN estimates of incidence and Mortality Worldwide for 36 cancers in 185 countries. CA Cancer J Clin.

[2] Xia Y, Li J, Liu G, Wang K, Qian G, Lu Z, Yang T, Yan Z, Lei Z, Si A (2020). Long-term Effects of repeat hepatectomy vs percutaneous radiofrequency ablation among patients with recurrent Hepatocellular Carcinoma: a Randomized Clinical Trial. JAMA Oncol.

[3] Kurebayashi Y, Matsuda K, Ueno A, Tsujikawa H, Yamazaki K, Masugi Y, Kwa WT, Effendi K, Hasegawa Y, Yagi H (2022). Immunovascular classification of HCC reflects reciprocal interaction between immune and angiogenic tumor microenvironments. Hepatology.

[4] Morse MA, Sun W, Kim R, He AR, Abada PB, Mynderse M, Finn RS (2019). The role of Angiogenesis in Hepatocellular Carcinoma. Clin Cancer Res.

[5] Tang W, Chen Z, Zhang W, Cheng Y, Zhang B, Wu F, Wang Q, Wang S, Rong D, Reiter FP (2020). The mechanisms of sorafenib resistance in hepatocellular carcinoma: theoretical basis and therapeutic aspects. Signal Transduct Target Ther.

[6] Garcia J, Hurwitz HI, Sandler AB, Miles D, Coleman RL, Deurloo R, Chinot OL (2020). Bevacizumab (Avastin(R)) in cancer treatment: a review of 15 years of clinical experience and future outlook. Cancer Treat Rev.

[7] Kopp F, Mendell JT (2018). Functional classification and experimental dissection of long noncoding RNAs. Cell.

[8] Tan YT, Lin JF, Li T, Li JJ, Xu RH, Ju HQ (2021). LncRNA-mediated posttranslational modifications and reprogramming of energy metabolism in cancer. Cancer Commun (Lond).

[9] Teng F, Zhang JX, Chang QM, Wu XB, Tang WG, Wang JF, Feng JF, Zhang ZP, Hu ZQ (2020). LncRNA MYLK-AS1 facilitates tumor progression and angiogenesis by targeting miR-424-5p/E2F7 axis and activating VEGFR-2 signaling pathway in hepatocellular carcinoma. J Exp Clin Cancer Res.

[10] Niu Y, Bao L, Chen Y, Wang C, Luo M, Zhang B, Zhou M, Wang JE, Fang YV, Kumar A (2020). HIF2-Induced Long Noncoding RNA RAB11B-AS1 promotes hypoxia-mediated angiogenesis and breast Cancer metastasis. Cancer Res.

[11] Tian C, Abudoureyimu M, Lin X, Chu X, Wang R (2021). Linc-ROR facilitates progression and angiogenesis of hepatocellular carcinoma by modulating DEPDC1 expression. Cell Death Dis.

[12] Yao RW, Wang Y, Chen LL (2019). Cellular functions of long noncoding RNAs. Nat Cell Biol.

[13] Li H, Wang S, Yao Q, Liu Y, Yang J, Xu L, Yang G (2021). A combined long noncoding RNA signature as a candidate Prognostic Biomarker for Ovarian Cancer. Front Oncol.

[14] Liu Z, Liu L, Weng S, Guo C, Dang Q, Xu H, Wang L, Lu T, Zhang Y, Sun Z (2022). Machine learning-based integration develops an immune-derived lncRNA signature for improving outcomes in colorectal cancer. Nat Commun.

[15] Li J, Chen Z, Tian L, Zhou C, He MY, Gao Y, Wang S, Zhou F, Shi S, Feng X (2014). LncRNA profile study reveals a three-lncRNA signature associated with the survival of patients with oesophageal squamous cell carcinoma. Gut.

[16] Newman AM, Liu CL, Green MR, Gentles AJ, Feng W, Xu Y, Hoang CD, Diehn M, Alizadeh AA (2015). Robust enumeration of cell subsets from tissue expression profiles. Nat Methods.

[17] Li Y, Jiang T, Zhou W, Li J, Li X, Wang Q, Jin X, Yin J, Chen L, Zhang Y (2020). Pan-cancer characterization of immune-related lncRNAs identifies potential oncogenic biomarkers. Nat Commun.

[18] Llovet JM, Castet F, Heikenwalder M, Maini MK, Mazzaferro V, Pinato DJ, Pikarsky E, Zhu AX, Finn RS (2022). Immunotherapies for hepatocellular carcinoma. Nat Rev Clin Oncol.

[19] Saad AM, Abdel-Megied AES, Elbaz RA, Hassab El-Nabi SE, Elshazli RM (2021). Genetic variants of APEX1 p.Asp148Glu and XRCC1 p.Gln399Arg with the susceptibility of hepatocellular carcinoma. J Med Virol.

[20] Guo L, Yi X, Chen L, Zhang T, Guo H, Chen Z, Cheng J, Cao Q, Liu H, Hou C (2022). Single-cell DNA sequencing reveals Punctuated and Gradual Clonal Evolution in Hepatocellular Carcinoma. Gastroenterology.

[21] Zhang MS, Cui JD, Lee D, Yuen VW, Chiu DK, Goh CC, Cheu JW, Tse AP, Bao MH, Wong BPY (2022). Hypoxia-induced macropinocytosis represents a metabolic route for liver cancer. Nat Commun.

[22] Lee HW, Xu Y, He L, Choi W, Gonzalez D, Jin SW, Simons M (2021). Role of venous endothelial cells in Developmental and Pathologic Angiogenesis. Circulation.

[23] Xu Z, Guo C, Ye Q, Shi Y, Sun Y, Zhang J, Huang J, Huang Y, Zeng C, Zhang X (2021). Endothelial deletion of SHP2 suppresses tumor angiogenesis and promotes vascular normalization. Nat Commun.

[24] Marchand M, Monnot C, Muller L, Germain S (2019). Extracellular matrix scaffolding in angiogenesis and capillary homeostasis. Semin Cell Dev Biol.

[25] Teichert M, Milde L, Holm A, Stanicek L, Gengenbacher N, Savant S, Ruckdeschel T, Hasanov Z, Srivastava K, Hu J (2017). Pericyte-expressed Tie2 controls angiogenesis and vessel maturation. Nat Commun.

[26] Gao S, Griffin CT (2021). RIPK3 modulates growth factor receptor expression in endothelial cells to support angiogenesis. Angiogenesis.

[27] Zakaria S, Helmy MW, Salahuddin A, Omran G (2018). Chemopreventive and antitumor effects of benzyl isothiocynate on HCC models: a possible role of HGF /pAkt/ STAT3 axis and VEGF. Biomed Pharmacother.

[28] Deng H, Kan A, Lyu N, Mu L, Han Y, Liu L, Zhang Y, Duan Y, Liao S, Li S (2020). Dual vascular endothelial growth factor receptor and fibroblast growth factor receptor inhibition elicits Antitumor Immunity and enhances programmed cell Death-1 checkpoint blockade in Hepatocellular Carcinoma. Liver Cancer.

[29] Liu DL, Lu LL, Dong LL, Liu Y, Bian XY, Lian BF, Xie L, Wen D, Gao DM, Ke AW (2020). Mir-17-5p and miR-20a-5p suppress postoperative metastasis of hepatocellular carcinoma via blocking HGF/ERBB3-NF-kappaB positive feedback loop. Theranostics.

[30] Zhao J, Li H, Zhao S, Wang E, Zhu J, Feng D, Zhu Y, Dou W, Fan Q, Hu J (2021). Epigenetic silencing of miR-144/451a cluster contributes to HCC progression via paracrine HGF/MIF-mediated TAM remodeling. Mol Cancer.

[31] Tong C, Liu H, Chen R, Zhu F (2021). The effect of TACE in combination with thalidomide-mediated adjuvant therapy on the levels of VEGF and bFGF in patients with hepatocellular carcinoma. Am J Transl Res.

[32] Lefere S, Van de Velde F, Hoorens A, Raevens S, Van Campenhout S, Vandierendonck A, Neyt S, Vandeghinste B, Vanhove C, Debbaut C (2019). Angiopoietin-2 promotes pathological angiogenesis and is a therapeutic target in Murine nonalcoholic fatty liver disease. Hepatology.

[33] Moeini A, Cornella H, Villanueva A (2012). Emerging signaling pathways in hepatocellular carcinoma. Liver Cancer.

[34] Statello L, Guo CJ, Chen LL, Huarte M (2021). Gene regulation by long non-coding RNAs and its biological functions. Nat Rev Mol Cell Biol.

[35] Qian X, Zhao J, Yeung PY, Zhang QC, Kwok CK (2019). Revealing lncRNA structures and interactions by sequencing-based approaches. Trends Biochem Sci.

[36] Lou Y, Yu Y, Xu X, Zhou S, Shen H, Fan T, Wu D, Yin J, Li G (2019). Long non-coding RNA LUCAT1 promotes tumourigenesis by inhibiting ANXA2 phosphorylation in hepatocellular carcinoma. J Cell Mol Med.

[37] Wang Y, Li W, Chen X, Li Y, Wen P, Xu F (2019). MIR210HG predicts poor prognosis and functions as an oncogenic lncRNA in hepatocellular carcinoma. Biomed Pharmacother.

[38] Chi J, Liu T, Shi C, Luo H, Wu Z, Xiong B, Liu S, Zeng Y (2019). Long non-coding RNA LUCAT1 promotes proliferation and invasion in gastric cancer by regulating miR-134-5p/YWHAZ axis. Biomed Pharmacother.

[39] Li ZY, Xie Y, Deng M, Zhu L, Wu X, Li G, Shi NX, Wen C, Huang W, Duan Y (2022). c-Myc-activated intronic miR-210 and lncRNA MIR210HG synergistically promote the metastasis of gastric cancer. Cancer Lett.

