# Supplementary material for: Novel risk model based on angiogenesis-related lncRNAs for prognosis prediction of hepatocellular carcinoma
Source: Cancer Cell Int. 2023 Aug 7;23:159. doi: 10.1186/s12935-023-02975-x (PMC10408211; doi:10.1186/s12935-023-02975-x)
Supplement: Supplementary file 1 — Figure S1. Macrophage infiltration in high and low risk groups. (A) Boxplots of differential distribution of macrophages in high- and low-risk groups across various databases. p<0.05. Figure S2. Gene set enrichment analysis (GSEA) of angiogenesis-related lncRNA classifiers. (A) Gene set enrichment analysis of high- and low-risk groups in the GO database. (B) Gene set enrichment analysis of high and low risk groups in the KEGG database GSEA, gene set enrichment analysis. When | NES | ≥ 1, FDR q-value <0.25 and NOM p-value < 0.01 were considered significant. Figure S3. (a) The association between voltage and the droplet diameter when the flow rate was 100 μl min-1; (b) The association between flow rate and the droplet diameter when the voltage was 6 kV; (c) The association between the inner flow rate (F1) and the droplet diameter when the outer flow rate F2 was 8 ml h-1; (d) The association between F2 and the droplet diameter when F1 was 0.4 ml h-1. Figure S4. Drug sensitivity screening for LUCAT1 and MIR210HG. Figure S5. Relative MIR210HG expression after using si-MIR210HG and sh-MIR210HG. [file 12935_2023_2975_MOESM1_ESM.docx]

**Supporting Information**

**Novel risk model based on angiogenesis-related lncRNAs for prognosis prediction of hepatocellular carcinoma**

Shicheng Xie ^1,#^, Jinwei Zhong ^1,#^, Zhongjing Zhang^2^, Weiguo Huang^2^, Xiaoben Lin^1^, Yating Pan^1^, Xiuyan Kong^1^,Hongping Xia^6^, Zhijie Yu^3,*^, Haizhen Ni^2,*^, Jinglin Xia^1,4,5, *^

*^1^Key Laboratory of Diagnosis and Treatment of Severe Hepato-Pancreatic Diseases of Zhejiang Province, The First Affiliated Hospital of Wenzhou Medical University, Wenzhou 325000, China*

*^2^Department of Vascular Surgery, The First Affiliated Hospital of Wenzhou Medical University, Wenzhou, Zhejiang Province, 325000, China*

*^3^Wenzhou Key Laboratory of Hematology, The First Affiliated Hospital of Wenzhou Medical University, Wenzhou, Zhejiang, China.*

*^4^Department of Interventional Radiology, The First Affiliated Hospital of Wenzhou Medical University, Wenzhou 325000, China*

*^5^Liver Cancer Institute, Zhongshan Hospital, Fudan University, Shanghai, 200032, China*

*^6^* *Zhongda Hospital, School of Medicine & Advanced Institute for Life and Health, Southeast University, Nanjing, 210009, China*

*Email: [zhijie_yu@wzhospital.cn](mailto:zhijie_yu@wzhospital.cn); [shanice0208@sina.com](mailto:shanice0208@sina.com); [xiajinglin@fudan.edu.cn](mailto:xiajinglin@fudan.edu.cn)

**^#^Note:** Xie S and Zhong J contributed equally to this work


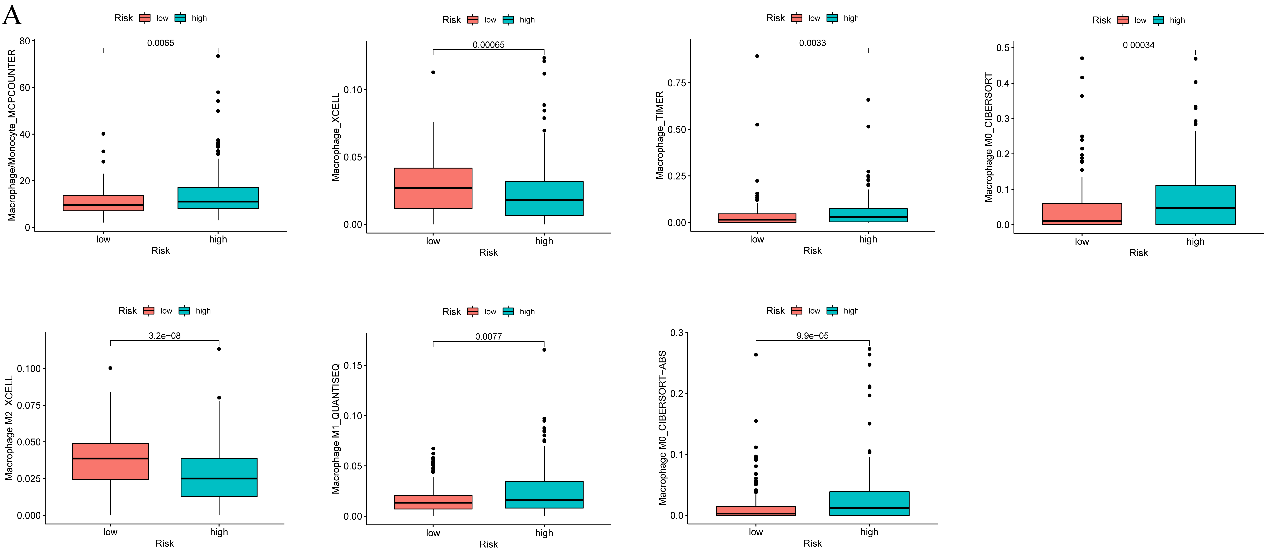


**Figure S1.** **Macrophage infiltration in high and low risk groups.** (A) Boxplots of differential distribution of macrophages in high- and low-risk groups across various databases. *p*<0.05.


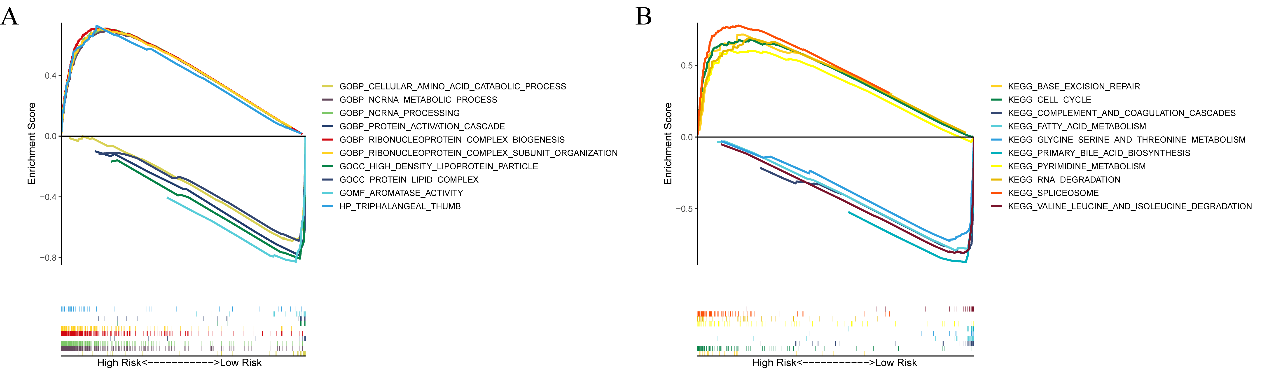


**Figure S2.** **Gene set enrichment analysis (GSEA) of angiogenesis-related lncRNA classifiers.** (A) Gene set enrichment analysis of high- and low-risk groups in the GO database. (B) Gene set enrichment analysis of high and low risk groups in the KEGG database GSEA, gene set enrichment analysis. When | NES | ≥ 1, FDR *q*-value <0.25 and NOM *p*-value <0.01 were considered significant.


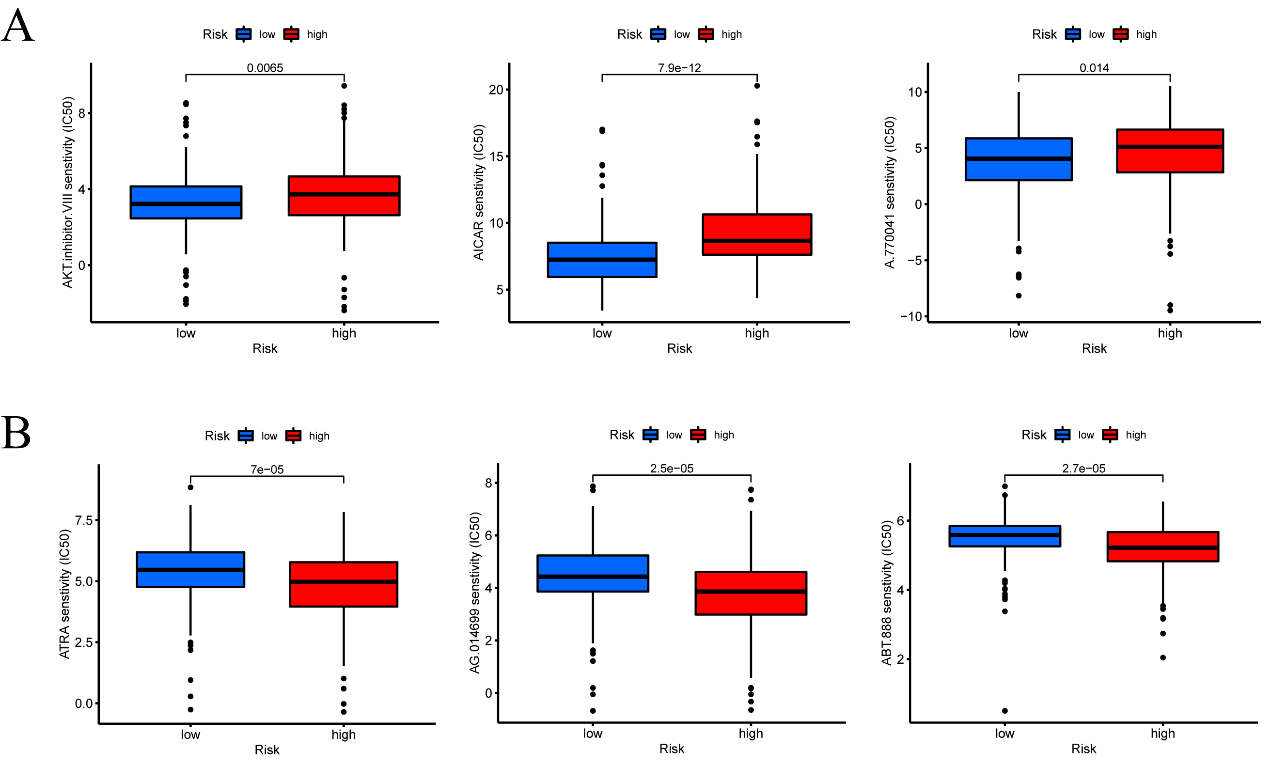


**Figure S3.** (a) The association between voltage and the droplet diameter when the flow rate was 100 μl min^-1^; (b) The association between flow rate and the droplet diameter when the voltage was 6 kV; (c) The association between the inner flow rate (*F*_1_) and the droplet diameter when the outer flow rate *F*_2_ was 8 ml h^-1^; (d) The association between *F*_2_ and the droplet diameter when *F*_1_ was 0.4 ml h^-1^.


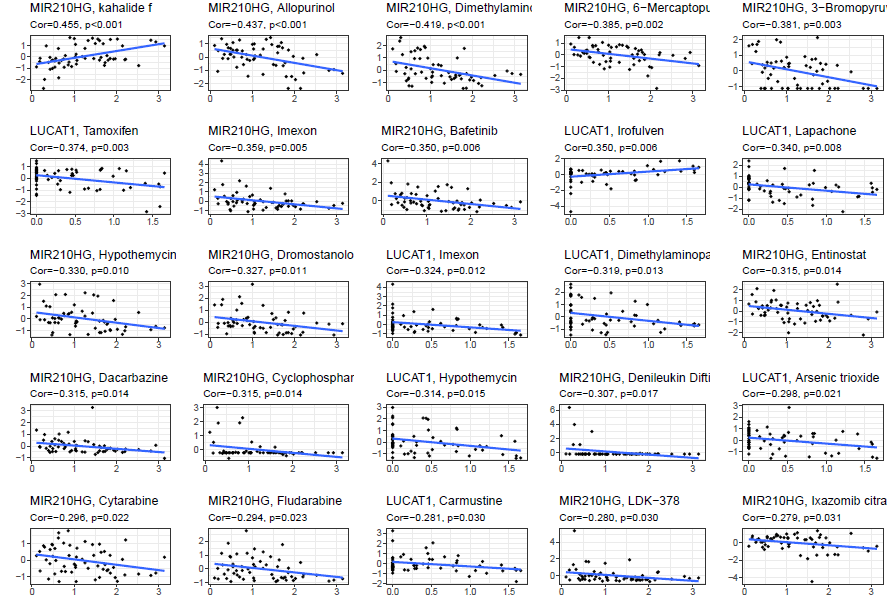


**Figure S4.** **Drug sensitivity screening for LUCAT1 and MIR210HG**.


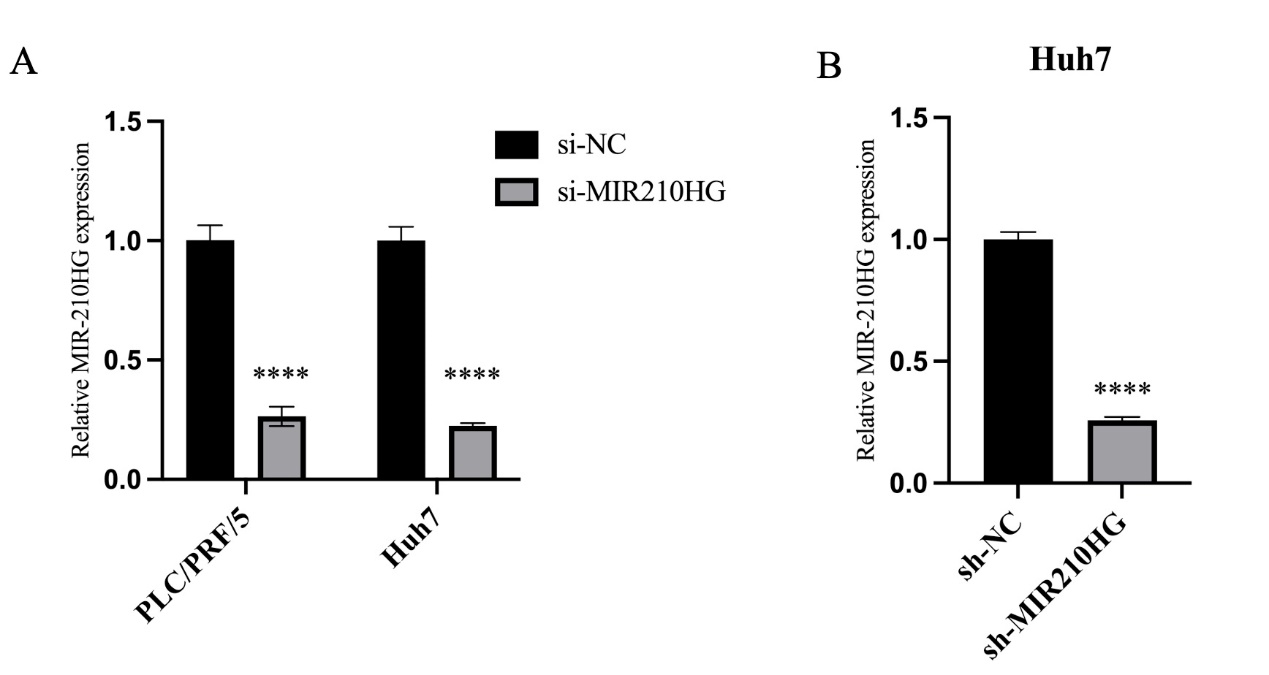


**Figure S5. Relative MIR210HG expression after using si-MIR210HG and sh-MIR210HG**.
